# Correction: Curcumin inhibits cellular condensation and alters microfilament organization during chondrogenic differentiation of limb bud mesenchymal cells

**DOI:** 10.1038/s12276-019-0365-5

**Published:** 2020-01-20

**Authors:** Dongkyun Kim, Song-Ja Kim, Shin-Sung Kang, Eun-Jung Jin

**Affiliations:** 1 Department of Biological Sciences, College of Natural Sciences, Wonkwang University, Iksan, 570-749 Korea; 2 Institute of Biotechnology, Wonkwang University, Iksan, 570-749 Korea; 3 Department of Biological Sciences, College of Natural Sciences, Kyungpook National University, Daegu, 702-701 Korea; 4 Department of Life Sciences, College of Natural Sciences, Kongju National University, Chungnam, 314-701 Korea

**Keywords:** Apoptosis, Cell signalling

**Correction to: Experimental & Molecular Medicine** 10.3858/emm.2009.41.9.072 Published online 29 May 2009

After online publication of this article, the authors noticed an error in the Fig. 1B, Fig. 3B, and Fig. 4A. We accidently added the wrong western bands in Fig. 1B, Fig. 3B, and Fig. 4A.

**Figure 1:**
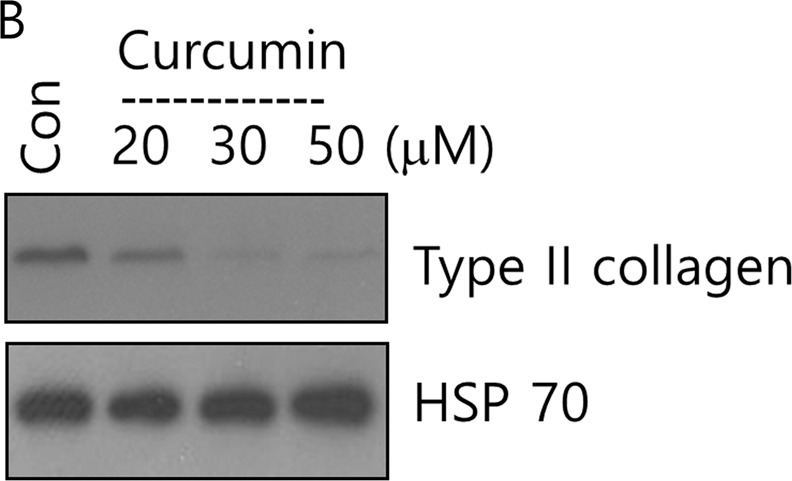
Fig. 1B

**Figure 2:**
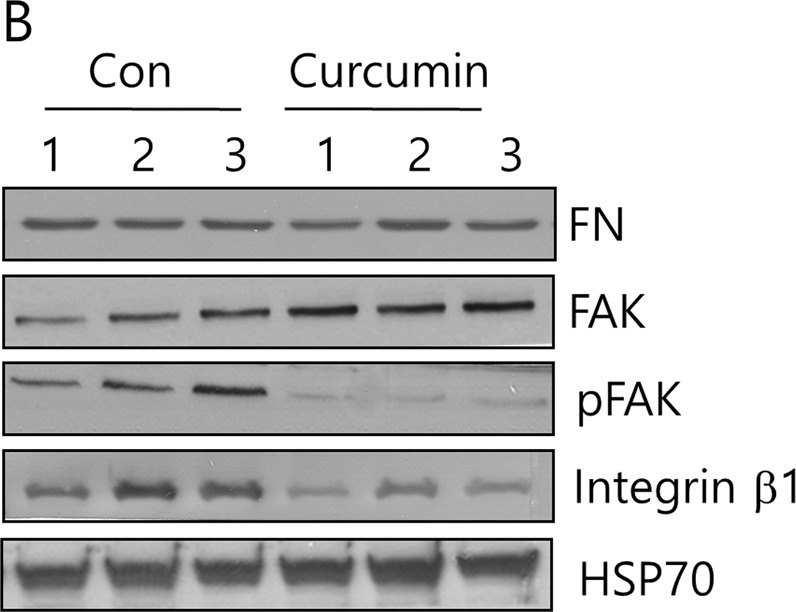
Fig. 3B

**Figure 3:**
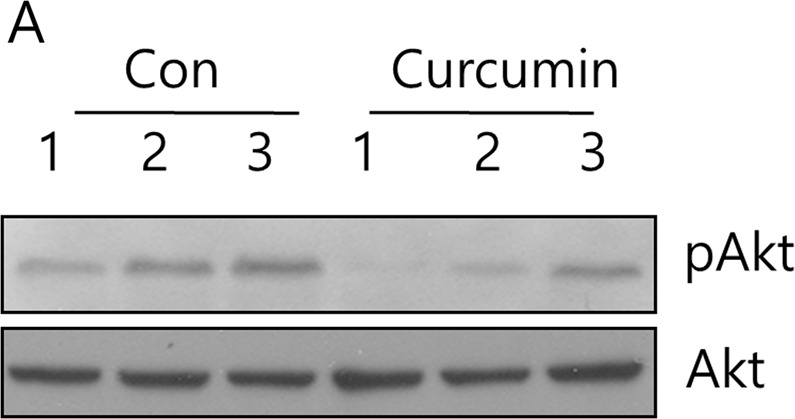
Fig. 4A

The authors apologize for any inconvenience caused.

